# Exploring the Relationship Between Inflammatory Biomarkers and Negative Symptoms Subtypes in Individuals at Ultra‐High Risk for Psychosis

**DOI:** 10.1111/eip.70183

**Published:** 2026-07-01

**Authors:** Dulari Hakamuwa Lekamlage, Ling Min Amelia Ang, Luba Sominsky, Stephen J. Wood, Patrick D. McGorry, Cristina Mei, G. Paul Amminger, Hok Pan Yuen, Melissa Kerr, Jessica Spark, Nick Wallis, Andrea Polari, Shelley Baird, Kate Buccilli, Sarah‐Jane A. Dempsey, Natalie Ferguson, Melanie Formica, Marija Krcmar, Amelia L. Quinn, Yohannes Mebrahtu, Arlan Ruslins, Rebekah Street, Cassandra Wannan, Lisa Dixon, Cameron S. Carter, Rachel Loewy, Tara A. Niendam, Martha Shumway, Barnarby Nelson, David Cotter, Subash Susai, Alison R. Yung

**Affiliations:** ^1^ Institute for Mental and Physical Health and Clinical Translation (IMPACT), School of Medicine Deakin University Geelong Australia; ^2^ Barwon Health Geelong Australia; ^3^ The University of Melbourne Parkville Australia; ^4^ University of Birmingham Birmingham UK; ^5^ Orygen Melbourne Australia; ^6^ Centre for Youth Mental Health The University of Melbourne Melbourne Australia; ^7^ Department of Psychiatry Columbia University New York New York USA; ^8^ University of California Irvine California USA; ^9^ Department of Psychiatry and Behavioral Sciences University of California San Francisco California USA; ^10^ Department of Psychiatry and Behavioral Sciences University of California, Davis Sacramento California USA; ^11^ Royal College of Surgeons in Ireland (RCSI) Dublin Ireland; ^12^ School of Health Sciences University of Manchester Manchester UK

## Abstract

**Aims:**

Negative symptoms are a core component of schizophrenia, affecting up to 60% of individuals with the disorder. They are categorised into primary negative symptoms (PNS), which are intrinsic to the illness, and secondary negative symptoms, which arise from external factors such as depression or medication side effects. They can also be divided into diminished expression and amotivation/anhedonia subtypes. While inflammation has been implicated in schizophrenia and linked to negative symptoms, little is known about whether inflammatory profiles differ between negative symptom subtypes in individuals at Ultra‐High Risk (UHR) for psychosis.

**Methods:**

We conducted a secondary analysis of 147 UHR participants from the Staged Treatment in Early Psychosis (STEP) study to examine whether inflammatory markers (Alpha‐2‐Macroglobulin, IL‐6, CRP, sICAM‐1, sVCAM‐1, and suPAR) differed across negative symptom subgroups, using multinomial and binomial logistic regression models adjusted for age, sex, smoking, and BMI.

**Results:**

Overall, most inflammatory markers were not significantly associated with negative symptom subgroups. However, higher sICAM‐1 levels were asscoiated with lower odds of primary negative symptoms compared with no negative symptoms. Additionally, younger age was associated with increased odds of PNS and amotivation, while smoking was associated with higher odds of secondary negative symptoms compared with no negative symptoms.

**Discussion:**

These findings suggest that inflammation may not broadly distinguish negative symptom subtypes at the UHR stage, although sICAM‐1 may play a role in early illness processes. Limitations include sample ascertainment, potential misclassification of negative symptoms, and the relatively small number of participants with PNS. Future studies with larger samples and longitudinal designs are needed to clarify whether inflammatory changes contribute to the emergence of specific negative symptom subtypes.

## 1 Introduction

Negative symptoms, such as low motivation and diminished facial expression, occur in up to 60% of individuals with schizophrenia (Correll and Schooler 2020), and are seen as a core component of the illness. There is now evidence of an association between inflammation and negative symptoms (Asevedo et al. 2014; El Kissi et al. 2015; Garcia‐Rizo et al. 2012). However, it is unclear how early inflammation begins in the trajectory of the illness. Thus, the objective of this research was to investigate inflammation and its association with negative symptoms in the Ultra High Risk (UHR) group. This group is at high risk of developing a psychotic disorder (Yung et al. 2004), with about 25% developing psychosis within 3 years of follow‐up (Salazar de Pablo et al. 2021).

A recent investigation of UHR individuals recruited into the Staged Treatment in Early psychosis (STEP) study revealed no association between the inflammatory markers measured at baseline with negative symptoms (data in preparation) (McGorry et al. 2023). However, this null finding may reflect limitations in treating negative symptoms as a unitary construct, rather than considering their distinct domains. Increasing evidence suggests that there are two core dimensions of negative symptoms: diminished expression (reduced facial and vocal expression, and reduced speech) and an experiential domain encompassing amotivation, apathy, asociality, and anhedonia (hereafter referred to as “amotivation”), and that these subgroups may have differing biological underpinnings (Strauss et al. 2013). Additionally, distinguishing between primary and secondary negative symptoms is crucial, as secondary symptoms (e.g., those due to depression, medication side effects, or positive symptoms) may obscure true associations with biological markers (Kirkpatrick et al. 2006). To address these limitations and better understand the heterogeneity of negative symptoms in UHR individuals, we conducted a secondary analysis of the STEP study to examine whether levels of inflammation differ across the different negative symptom subgroups of diminished expression and amotivation, and primary and secondary negative symptoms. Details of the study design and measures are provided in the Methods section.

### 1.1 Negative Symptoms in Schizophrenia

While negative symptoms can appear at any time in the course of psychotic disorders, they are often one of the first symptoms reported (Correll and Schooler 2020). Negative symptoms consist of two subtypes: diminished expression and amotivation (Foussias et al. 2014). Negative symptoms can also be categorised into primary and secondary negative symptoms (Mosolov and Yaltonskaya 2021). Primary negative symptoms (PNS) are symptoms that are intrinsic to the pathophysiology of the illness itself, whereas secondary negative symptoms are symptoms that can arise as a result of environmental and comorbidity factors such as prolonged hospitalisation, side‐effects of anti‐psychotics, depression, and substance abuse (Mosolov and Yaltonskaya 2021). While secondary negative symptoms can be improved once these factors are removed, primary negative symptoms remain difficult to treat as they do not respond well to currently available anti‐psychotic medications (Correll and Schooler 2020). Negative symptoms are one of the strongest predictors of poor functional outcomes in individuals with schizophrenia (Correll and Schooler 2020). It is therefore important to find new treatments to treat these symptoms in order to improve outcomes.

### 1.2 Inflammation is Linked to Negative Symptoms in Schizophrenia

Inflammation is a physiological, protective response to an underlying infectious or non‐infectious process. It involves cells of the innate immune system, adaptive immune system, and inflammatory mediators such as cytokines. Inflammatory responses are beneficial if, as is usually the case, they stop with the destruction of the injurious agents that initiated them. However, inflammatory responses are maladaptive if they are self‐directed, too extreme, or persist, resulting in pathology from chronic inflammation (Oronsky et al. 2022).

Chronic inflammation is thought to be one of the mechanisms underlying the pathophysiology of schizophrenia (Miller and Goldsmith 2017). Meta‐analyses have reported an increased level of inflammatory markers in individuals with schizophrenia (Goldsmith et al. 2016; Miller et al. 2011; Potvin et al. 2008), and there is a growing number of studies linking higher levels of inflammatory markers to negative symptoms in both chronic and recent‐onset schizophrenia (Asevedo et al. 2014; El Kissi et al. 2015; Garcia‐Rizo et al. 2012). A 2022 systematic review and meta‐analysis found a significant association between the pro‐inflammatory cytokines IL‐1*β*, IL‐2, IL‐6 and TNF‐*α* and negative symptoms in first episode psychosis, and a negative association with IL10, considered to be an anti‐inflammatory cytokine. However, a positive association was also found between negative symptoms and IL‐4, also thought to be anti‐inflammatory (Dunleavy et al. 2022). Higher levels of circulating inflammatory markers have also been found in individuals with deficit syndrome schizophrenia (Garcia‐Rizo et al. 2012; Goldsmith et al. 2018), a subgroup of schizophrenia in which primary negative symptoms are persistent (Goldsmith et al. 2018). For example, the pro‐inflammatory cytokines IL‐6 (Garcia‐Rizo et al. 2012; Goldsmith et al. 2019) and TNF‐*α* have been found to be associated with deficit syndrome schizophrenia as has the inflammatory marker CRP compared to the non‐deficit group. Neuroinflammation has also been implicated in structural brain abnormalities (Williams et al. 2022), with higher levels of IL‐6 being associated with greater grey matter volume in temporal regions (including the middle and inferior temporal cortex, fusiform gyrus), but reduced grey matter volume and reduced cortical thickness in frontal regions. Such structural brain abnormalities have been found in patients with deficit schizophrenia (Fischer et al. 2012; Kanahara et al. 2013). Thus, it seems that there may be an association between inflammation and negative symptoms.

Alpha‐2‐macroglobulin (A2M) is a protease inhibitor, that is, it blocks the action of proteolytic enzymes from breaking down proteins. A2M is released during inflammation to prevent tissue damage as a result of proteolytic activity (Hashim 2024). Consistent with this mechanism, low levels of A2M have been found to be associated with increased risk of development of psychosis in the UHR population (English et al. 2018; Mongan et al. 2023). However, there are no studies of the association between A2M and negative symptoms.

ICAM‐1 and VCAM‐1 are, respectively, endothelial and immune cell adhesion proteins that are upregulated in response to pro‐inflammatory stimuli. These molecules play a key role in leukocyte transmigration and are integral to maintaining the structural and functional integrity of the blood–brain barrier (BBB) and blood–cerebrospinal fluid barrier (BCSFB) (Meixensberger et al. 2021). Soluble intercellular adhesion molecule‐1 (sICAM‐1) and soluble vascular cell adhesion molecule‐1 (sVCAM‐1) are circulating forms of ICAM‐1 and VCAM‐1. Elevated levels of sICAM‐1 and sVCAM‐1 in serum and cerebrospinal fluid (CSF) have been reported in individuals with schizophrenia, suggesting an underlying pro‐inflammatory state and potential disruption of neurovascular barriers (Meixensberger et al. 2021; Schwarz et al. 2000; Sheikh et al. 2023).

sICAM‐1 and sVCAM‐1 may offer insight into early pathophysiological changes. While most evidence comes from chronic schizophrenia samples, lower sICAM‐1 levels have also been observed in early‐stage and unmedicated patients (Muller 2019; Schwarz et al. 2000). These molecules have also been associated with clinical features such as negative symptoms, although findings are inconsistent. Some studies report positive associations, while others, particularly those with younger or early‐phase cohorts, do not find such links (Schwarz et al. 2000; Sheikh et al. 2023). Notably, studies that observed associations between sICAM‐1 and negative symptoms often did not distinguish between primary and secondary negative symptoms and included participants with relatively high mean age (around 33) or potential comorbidities like cardiovascular disease, which independently raise sICAM‐1 levels (Muller 2019; Rohde et al. 1999).

Given their roles in neurovascular integrity and immune activation and their possible link with negative symptoms in early psychosis and medication naïve patients with schizophrenia, sICAM‐1 and sVCAM‐1 may be biologically relevant biomarkers to investigate in UHR populations. Measuring their levels at this early stage of illness could help clarify whether CAM dysregulation precedes the onset of psychosis and whether it is related to negative symptoms.

Soluble urokinase plasminogen activator receptor (suPAR) is a marker of systemic inflammation that is expressed upon immune activation. It has pro‐inflammatory properties and positively correlates with known and established pro‐inflammatory markers such as CRP, IL‐6, and TNF‐*α* (Rasmussen et al. 2021). suPAR has a long plasma half‐life of approximately 7–10 days (Velissaris et al. 2021), suggesting that it does not degrade easily. This offers a significant advantage in using suPAR as a biomarker since most cytokines have shorter half‐lives (Donnelly et al. 2009), which can lead to inaccurate measurements and inconsistent findings.

### 1.3 Inflammation in Ultra‐High Risk (UHR) Individuals

It is not clear how early in the illness course these inflammatory processes emerge. Evidence suggests that high levels of inflammation may already be present in those at UHR for psychosis (Aymerich et al. 2025; Misiak et al. 2021; Park and Miller 2020). Meta‐analyses have reported altered inflammatory profiles in UHR individuals compared with healthy controls (Misiak et al. 2021; Park and Miller 2020).

Most studies to date have focused on peripheral inflammatory markers (e.g., circulating cytokines). Among these, IL‐6 has been the most consistently elevated in UHR individuals (Misiak et al. 2021; Mondelli et al. 2023; Park and Miller 2020). However, findings across other cytokines are inconsistent (Aymerich et al. 2025; Bloomfield et al. 2016; Di Biase et al. 2017; Hafizi et al. 2017). In contrast, a smaller number of studies have examined central inflammation, primarily using PET imaging of microglial activation or CSF markers. For example, Bloomfield et al. reported increased microglial activation in UHR individuals (Bloomfield et al. 2016), an indicator of neuroinflammation (Woodburn et al. 2021). However, other PET studies found no differences in microglial activity between UHR individuals and healthy controls (Di Biase et al. 2017; Hafizi et al. 2017).

It is important to note that findings from peripheral blood studies and central measures (PET, CSF) are not directly comparable. While systemic and central inflammation may be linked, they reflect distinct biological processes, and elevations in blood cytokines do not necessarily imply parallel changes in the brain.

To date, only two studies have directly examined the relationship between inflammation and negative symptoms in the UHR group (Goldsmith et al. 2019; Ye et al. 2023). Both reported that higher levels of peripheral TNF were associated with more severe negative symptoms. This suggests that peripheral inflammation may contribute to the development of negative symptomatology, although the role of central neuroinflammation in this association remains unclear.

### 1.4 Gaps in Literature

Although there is some evidence that inflammation is linked to negative symptoms in schizophrenia, early psychosis, and UHR individuals (Asevedo et al. 2014; El Kissi et al. 2015; Garcia‐Rizo et al. 2012; Goldsmith et al. 2019; Ye et al. 2023), results are not consistent. Additionally, there are no studies that assessed potential differences in inflammation between the different subtypes of negative symptoms. Therefore, the aim of this paper was to determine if levels of inflammatory markers differed between primary and secondary negative symptoms, and between diminished expression and amotivation subtypes.

## 2 Methods

### 2.1 Study Design

This study is a secondary analysis of data from the STEP study (McGorry et al. 2023). The primary aim of the STEP study was to examine the functional outcomes of UHR patients following a sequential intervention strategy which consisted of Support and Problem Solving (SPS), Cognitive‐Behavioural Case Management (CBCM), and antidepressant medication. As a secondary outcome, bloods were taken at baseline and at different stages of the sequential intervention strategy for analyses. A total of 342 participants were recruited to the study. Of the 342 participants, only 147 participants had baseline data on inflammatory marker scores and potential confounders (e.g., body mass index (BMI, kgm^−2^), smoking, age, and sex). Negative symptoms were assessed using the Scale for the Assessment of Negative Symptoms (SANS) (Kumari et al. 2017) and positive symptoms were assessed using the Brief Psychiatric Rating Scale (BPRS) (Lyne et al. 2012). The participants' demographic details and inflammatory markers levels are presented in Table 1. Within the negative symptom domain, 12 participants belonged to both the primary negative symptom group and the amotivation group.

**TABLE 1:** Demographic data of patients in the STEP study.

	Total sample *N* = 147	No negative symptoms *N* = 78	Primary negative symptoms *N* = 15	Secondary negative symptoms *N* = 54	Amotivation *N* = 62	No amotivation *N* = 85
Males (%)	56 (38)	34 (43.6)	7 (46.7)	15 (27.8)	20 (32.3)	36 (42.2)
Mean age (SD)	23.3 (2.5)	23.7 (2.8)	22.1 (2)	23.1 (2.2)	23 (2.3)	23.5 (2.8)
Mean BMI (SD)	25.7 (5.9)	25.9 (6.0)	25.5 (7.8)	25.4 (5.1)	25.7 (5.6)	25.7 (6.1)
Smoking status: yes (%)	80 (54.4)	37 (47.4)	9 (60)	34 (63)	40 (64.5)	40 (47.1)
Mean A2M (SD)	2119.6 (585.3)	2047.7 (586)	2093.9 (430.5)	2234.1 (618)	2196.8 (588.2)	2073.7 (581.8)
Mean log (IL‐6) (SD)	−1.1 (0.7)	−1.1 (0.7)	−1.1 (0.7)	−1 (0.7)	−1 (0.6)	−1.1 (0.8)
Mean log (CRP) (SD)	13.4 (1.4)	13.4 (1.5)	13.6 (1.5)	13.5 (1.3)	13.5 (1.3)	13.4 (1.5)
Mean log (sICAM‐1) (SD)	12.7 (0.2)	12.7 (0.2)	12.6 (0.1)	12.7 (0.2)	12.7 (0.2)	12.7 (0.2)
Mean log (sVCAM‐1) (SD)	12.9 (0.2)	12.9 (0.2)	13 (0.2)	12.9 (0.2)	12.9 (0.2)	12.9 (0.2)
Mean log (suPAR) (SD)	0.9 (0.2)	0.9 (0.2)	1 (0.3)	0.8 (0.3)	0.9 (0.3)	0.9 (0.3)

Abbreviations: A2M (Alpha‐2‐macroglobulin); BMI: body mass index; CRP (C‐reactive protein); IL‐6 (interleukin 6); sICAM‐1 (Soluble intercellular adhesion molecule‐1); suPAR: soluble urokinase plasminogen activator receptor; sVCAM‐1 (Soluble vascular cell adhesion protein 1).

### 2.2 Participants

Participants were primarily recruited from youth mental health services such as the Headspace centres that are funded through the Commonwealth Government of Australia (McGorry et al. 2023). These centres are located in purpose‐built premises in shopping and community precincts in the suburbs of Sunshine, Glenroy, Werribee, and Craigieburn, in the North Western Melbourne metropolitan region in the state of Victoria, Australia, and provide access to primary care, as well as mental health and welfare services for patients aged 12–25 years old.

UHR status was determined using a 2‐step process. In the first step, the Prodromal Questionnaire‐16 (PQ‐16) was administered (Ising et al. 2012). Then, individuals who obtained a score of 6 or above for positive symptoms on the PQ‐16 underwent further assessment using the standardised Comprehensive Assessment of At‐Risk Mental States (CAARMS) (Yung et al. 2005) and the Social and Occupational Functioning Assessment Scale (SOFAS) (Goldman et al. 1992) to confirm UHR status. A cut‐off score of 6 on the PQ‐16 has been shown to identify individuals at ultra‐high risk for psychosis effectively as it has a high sensitivity and specificity of 87% (Lim et al. 2009; Phillips et al. 2009). While the UHR criteria have been shown to have good specificity for prediction of psychosis (Webb et al. 2015; Woods et al. 2018), rates of comorbidity at baseline are high, especially for non‐psychotic disorders such as depression. There was therefore the need to distinguish primary and secondary negative symptoms in this study. See below for details.

### 2.3 Inclusion and Exclusion Criteria

#### 2.3.1 Inclusion Criteria

A participant was considered eligible for this study only if all the following criteria apply:

1. Aged between 12 and 25 years during the time of entry
2. Sufficient English proficiency to undertake assessments
3. Ability to give informed consent. For minors below the age of 18, consent was given from both a parent/legal guardian, as well as the participants themselves.
4. Meeting criteria for at least one UHR group (Yung et al. 2003).
  - “Attenuated Psychotic Symptoms” individuals have “subthreshold” positive symptoms that deviate from normal phenomena but which are not yet frankly psychotic,
  - “Brief Limited Intermittent Psychotic Symptoms (BLIPS)” individuals have symptoms of psychotic intensity, but which are very infrequent, or which have a total duration of less than 7 days before resolving spontaneously.
  - “Trait and State Risk Factors” individuals have a trait risk factor for psychotic disorder such as having schizotypal personality disorder or a family history of a psychotic disorder in a first‐degree relative, plus a marked deterioration in functioning of at least 1 month in duration. This severity criterion is necessary to exclude otherwise normal relatives of patients with psychotic illnesses who have a brief period of mild symptoms.

#### 2.3.2 Exclusion Criteria

1. Having a history of a psychotic episode that lasted for a week or more, regardless of whether antipsychotic medications had been taken or not.
2. Attenuated psychotic symptoms only appeared during acute intoxication with substances.
3. Having an organic brain disease for example, temporal lobe epilepsy, that is known to lead to psychotic symptoms.
4. Suffering from any illness (metabolic/endocrine/physical), for example, thyroid disease, that is known to cause neuropsychiatric consequences.
5. Being diagnosed with a severe developmental disorder, for example, Severe Autism Spectrum Disorder.
6. Having a history of developmental or intellectual delay/disability.
7. Having or had a previous or current SCID diagnosis of bipolar disorder I.
8. More details about sample ascertainment and recruitment methods can be found in (McGorry et al. 2023). The use of antipsychotic/antidepressant medication currently or in the past did not serve as a reason for exclusion. If participants were using antipsychotic or antidepressant medication at entry to the study, the medication was tapered and ceased. This approach aligns with clinical guidelines as antipsychotics are not recommended for the UHR population, and antidepressants for young individuals are suggested only in cases of severe depression, whereas psychosocial interventions are suggested for those with mild to moderate depression (Bernstein et al. 1997; McDermott et al. 2011; van der Gaag et al. 2013). The majority of UHR individuals tend to suffer from mild to moderate depression, rather than severe (Fusar‐Poli et al. 2014; Phillips et al. 2009).

### 2.4 Biospecimens

Blood samples were collected between 8:00 am and 10:00 am at the local pathology laboratory. Participants were instructed to fast on the morning of collection. Whole blood was collected in 10 mL EDTA tubes and processed at the collection site shortly after sampling to obtain plasma. Briefly, samples were kept on ice and centrifuged within approximately 90 min of collection (1000–2500 × g for 10 min at room temperature). The separated plasma was aliquoted and stored at −80°C at the University of Queensland and the University of Adelaide. The quantification of immune‐biomarkers was conducted at the Royal College of Surgeons in Ireland. Peripheral blood samples were obtained from the participants at baseline and 6‐month follow‐up. Plasma levels of IL‐6, CRP, sICAM‐1, sVCAM‐1 and suPAR were measured using the Pro‐inflammatory Panel 1, Cytokine Panel 1 and Vascular Injury Panel 2 v‐PLEX multiplex immunoassay kits (Mesoscale Discovery Systems) according to the manufacturer's instructions. A Sector Imager 2400 plate reader was used to quantify concentrations of each marker (Meso‐Scale Diagnostics). A2M and suPAR were quantified using Abcam and Virogates suPARnostic ELISA kits according to manufacturer's instructions.

### 2.5 Negative Symptom Subtypes

We were interested in investigating inflammatory biomarkers associated with primary negative symptoms compared to secondary negative symptoms and in the amotivation subtype compared to the diminished expression subtype. To do this, new negative symptoms variables were created using IBM SPSS Statistics 29.0 as follows:

Presence of primary negative symptoms was defined as having low depression, low positive symptoms, and high negative symptoms, and calculated as follows: a combined total score of ≤ 6 on the BPRS subscales of depression, suicidality, and guilt (corresponding to an average score of ‘very mild’ on each of these items), and a combined total score of 16 or less on the BPRS psychotic subscales of suspiciousness, hallucinations, unusual thought content, and conceptual disorganisation (corresponding to a score of ‘moderate’ on each of these items) and a SANS score of ≥ 3 for Global Rating of Affective Flattening OR Global Rating of Alogia OR Global Rating of Avolition/Apathy OR Global Rating of Anhedonia/Asociality OR Global Rating of Attention (Yung et al. 2019).

Presence of secondary negative symptoms was defined as having negative symptoms and either high depression or high positive symptoms and calculated as follows: a combined total score of > 6 on the BPRS subscales of depression, suicidality and guilt, or a combined total score > 16 on the BPRS psychotic subscales of suspiciousness, hallucinations, unusual thought content and conceptual disorganisation and a SANS score of ≥ 3 for Global Rating of Affective Flattening OR Global Rating of Alogia OR Global Rating of Avolition/Apathy OR Global Rating of Anhedonia/Asociality OR Global Rating of Attention (Yung et al. 2019).

Amotivation was defined by having a SANS score of ≥ 3 for SANS Avolition/Apathy OR Anhedonia/Asociality and diminished expression was defined by having a SANS score of ≥ 3 for SANS Affective Flattening OR Alogia (Strauss et al. 2013). However, there was only a very small proportion of participants (19, 12.8%) who met criteria for diminished expression. Therefore, we decided to create two variables: (1) No amotivation group (which includes participants with diminished expression only, as well as participants with neither diminished expression nor amotivation) and (2) Amotivation group (which includes participants with amotivation, as well as participants with amotivation and diminished expression).

Any individuals with a SANS score of < 3 for Global Rating of Affective Flattening OR Global Rating of Alogia OR Global Rating of Avolition/Apathy OR Global Rating of Anhedonia/Asociality OR Global Rating of Attention were considered as not having any negative symptoms.

### 2.6 Confounders

The following a priori confounders were considered:

*Smoking* is associated with oxidative stress, which in turn leads to an inflammatory response (Rahman and Adcock 2006). This was assessed using the Alcohol, Smoking, and Substance Involvement Screening Test (ASSIST) (Caliri et al. 2021). *Higher body mass index* is also associated with inflammation (Fernandez‐Sanchez et al. 2011), and was assessed using patient's height and weight information. *Age* is positively correlated with inflammation (Chung et al. 2019). *Biological sex* influences immune responses, with females generally exhibiting stronger immunity than males, which can affect susceptibility to infections, inflammatory conditions, and subsequent risk of neuropsychiatric disorders (Klein and Flanagan 2016).

### 2.7 Statistical Analyses

This was a cross‐sectional analysis conducted on baseline data. Statistical analyses were conducted using IBM SPSS Statistics 29.0 (IBM Corporation 2023). Multinomial and binomial logistic regression were used to investigate the effect of inflammatory markers on the likelihood of belonging to the negative symptom subtypes. Multinomial logistic regression models for negative symptoms were fitted with two reference categories: no negative symptoms (Table 2) and secondary negative symptoms (Table 3). For amotivation subtypes, binary logistic regression models were fitted with no amotivation as the reference level, adjusting for depression (derived from the BPRS subscales: depression, suicidality, and guilt) (Table 4). In all models, the six inflammatory markers were used as independent variables. IL‐6, CRP, sICAM‐1, sVCAM‐1, and suPAR exhibited positively skewed distributions and were therefore log‐transformed prior to analysis to improve distributional symmetry and reduce the influence of extreme values. A2M was analysed on its original scale. Models were adjusted for age, sex, smoking status, and BMI (log scaled). To account for multiple testing across biomarkers, *p* were adjusted using the Benjamini–Hochberg (BH) procedure within each model/contrast.

## 3 Results

The sample consisted of 147 individuals meeting the UHR criteria, all recruited to the STEP study. The mean age of the sample was 23.3 years (SD 2.5); 38% of the sample were males. Markers of inflammation and levels of potential confounder for the sample can be found in Table 1.

### 3.1 Inflammatory Markers and Negative Symptoms

Higher levels of sICAM‐1 were significantly associated with lower odds of having primary negative symptoms compared to both reference levels; no negative symptoms (Table 2) and secondary negative symptoms (Table 3); however, after adjustment for multiple testing, statistical significance was retained only for the comparison with no negative symptoms. The odds of having primary negative symptoms (versus not having negative symptoms) were 99.8% lower for each one‐unit increase in log‐transformed sICAM‐1 (Odds ratio (OR) = 0.002, 95% CI: 2.426e‐5–0.154, adj. *p* = 0.03) (Table 2), corresponding to an approximately 2.72‐fold increase in the original sICAM‐1 concentration. There was no difference in sICAM‐1 level between the no negative symptoms group and the secondary negative symptoms group. sICAM‐1 was not associated with presence of amotivation (Table 4). No other inflammatory markers were associated with any negative symptom subtype.

**TABLE 2:** Multinomial logistic regression results for negative symptoms subtypes considering not having negative symptoms subtype as the reference level.

Predictor	Primary negative symptoms versus no negative symptoms						Secondary negative symptoms versus no negative symptoms					
	Estimate (B)	(SE)	*p*	BH adj. *p*	Odds ratio (OR)	95% CI for OR	Estimate (B)	(SE)	*p*	BH adj. *p*	Odds ratio (OR)	95% CI for OR
Intercept	44.358	27.098	0.102	—	N/A	N/A	8.996	15.053	0.550	—	N/A	N/A
Sex = male	0.225	0.713	0.752	—	1.253	0.310, 5.063	−0.577	0.428	0.177	—	0.562	0.243, 1.299
Age	−0.480	0.189	**0.011**	—	0.619	0.428, 0.896	−0.153	0.087	0.080	—	0.858	0.724, 1.018
Log (BMI)	−1.249	1.775	0.482	—	0.287	0.009, 9.309	−0.332	1.147	0.772	—	0.718	0.076, 6.790
Smoking = yes	0.774	0.691	0.262	—	2.168	0.560, 8.395	0.995	0.420	**0.018**	—	2.704	1.187, 6.161
Biomarkers												
A2M	0.000	0.001	0.760	0.760	1.000	0.999, 1.001	0.000	0.000	0.226	0.896	1.000	1.000, 1.001
Log (IL‐6)	−0.251	0.579	0.664	0.760	0.778	0.250, 2.420	0.027	0.354	0.940	0.940	1.027	0.513, 2.056
Log (CRP)	0.565	0.295	0.056	0.168	1.759	0.987, 3.135	0.145	0.192	0.448	0.896	1.157	0.794, 1.684
Log (sICAM‐1)	−6.250	2.233	0.005	**0.030**	0.002	2.426e‐5, 0.154	−0.474	1.134	0.676	0.940	0.623	0.067, 5.750
Log (sVCAM‐1)	2.986	1.930	0.122	0.244	19.809	0.450, 871.059	−0.109	0.959	0.910	0.940	0.897	0.137, 5.875
Log (suPAR)	1.449	1.369	0.290	0.435	4.257	0.291, 62.266	−0.610	0.780	0.435	0.896	0.544	0.118, 2.508

*Note:* Bold values indicate statistical significance at *p* < 0.05.

Abbreviations: A2M (Alpha‐2‐macroglobulin); BMI: body mass index; CRP (C‐reactive protein); IL‐6 (interleukin 6); sICAM‐1 (Soluble intercellular adhesion molecule‐1); suPAR: soluble urokinase plasminogen activator receptor; sVCAM‐1 (Soluble vascular cell adhesion protein 1).

**TABLE 3:** Multinomial logistic regression results for negative symptoms subtype considering secondary symptoms subtype as the reference level.

Predictor	Primary negative symptoms versus secondary negative symptoms					
	Estimate (B)	(SE)	*p*	BH adj. *p*	Odds ratio (OR)	95% CI for OR
Intercept	35.362	27.398	0.197	—	N/A	N/A
Sex = male	0.802	0.734	0.275	—	2.230	0.529, 9.404
Age	−0.327	0.189	0.084	—	0.721	0.498, 1.045
Log (BMI)	−0.917	1.806	0.612	—	0.400	0.012, 13.764
Smoking = yes	−0.221	0.709	0.755	—	0.802	0.200, 3.218
Biomarkers						
A2M	−0.001	0.001	0.342	0.410	0.999	0.998, 1.001
Log (IL‐6)	−0.278	0.583	0.633	0.633	0.757	0.241, 2.376
Log (CRP)	0.419	0.296	0.157	0.236	1.521	0.850, 2.719
Log (sICAM‐1)	−5.776	2.265	0.011	0.066	0.003	3.658e‐5, 0.263
Log (sVCAM‐1)	3.095	1.934	0.110	0.236	22.089	0.499, 978.224
Log (suPAR)	2.058	1.405	0.143	0.236	7.832	0.498, 123.070

Abbreviations: A2M (Alpha‐2‐macroglobulin); BMI: body mass index; CRP (C‐reactive protein); IL‐6 (interleukin 6); sICAM‐1 (Soluble intercellular adhesion molecule‐1); suPAR: soluble urokinase plasminogen activator receptor; sVCAM‐1 (Soluble vascular cell adhesion protein 1).

**TABLE 4:** Amotivation Versus no amotivation.

Predictor	Estimate (B)	(SE)	*p*	BH adj. *p*	Odds ratio (OR)	95% CI for OR
Intercept	8.299	16.058	0.605	—	N/A	N/A
Sex = male	0.248	0.445	0.577	—	1.282	(0.536, 3.063)
Age	−0.227	0.093	**0.015**	—	0.797	(0.664, 0.957)
Log (BMI)	0.741	1.186	0.532	—	2.098	(0.205, 21.432)
Smoking = Yes	0.663	0.439	0.131	—	1.941	(0.820, 4.592)
Depression	0.351	0.075	**< 0.001**	—	1.421	(1.226, 1.646)
Biomarkers						
A2M	0.000	0.000	0.366	0.549	1.000	(1.000, 1.001)
Log (IL‐6)	−0.116	0.366	0.751	0.901	0.890	(0.435, 1.823)
Log (CRP)	0.230	0.191	0.230	0.549	1.258	(0.865, 1.831)
Log (sICAM‐1)	−2.013	1.151	0.080	0.480	0.134	(0.014, 1.274)
Log (sVCAM‐1)	0.954	0.985	0.333	0.549	2.596	(0.376, 17.913)
Log (suPAR)	0.014	0.806	0.986	0.986	1.014	(0.209, 4.923)

*Note:* Bold values indicate statistical significance at *p* < 0.05.

Abbreviations: A2M (Alpha‐2‐macroglobulin); BMI: body mass index; CRP (C‐reactive protein); IL‐6 (interleukin 6); sICAM‐1 (Soluble intercellular adhesion molecule‐1); suPAR: soluble urokinase plasminogen activator receptor; sVCAM‐1 (Soluble vascular cell adhesion protein 1).

### 3.2 Association of Confounders With Negative Symptoms

Age was inversely associated with primary negative symptoms compared to no negative symptoms (OR: 0.619, 95% CI: 0.428–0.896, *p* = 0.011), indicating that younger individuals had higher odds of primary negative symptoms (Table 2). Similarly, younger age was associated with higher odds of amotivation (OR: 0.797, 95% CI: 0.664–0.957, *p* = 0.015) (Table 4). In addition, higher depression scores were associated with increased odds of amotivation (OR: 1.421, 95% CI: 1.226–1.646, *p* < 0.001). Smoking status was associated with higher odds of secondary negative symptoms compared to no negative symptoms (OR: 2.704, 95% CI: 1.187–6.161, *p* = 0.018) (Table 2).

### 3.3 Symptom‐Level Comparisons Between STEP and PACE Participants

To explore whether differences in sample ascertainment of our UHR group could explain the lack of association between negative symptoms and inflammatory markers, we conducted a post hoc analysis comparing symptom severity between cohorts. STEP participants were primarily recruited from primary care settings (Headspace), whereas earlier Australian UHR studies (e.g., PACE Clinic) recruited from tertiary mental health services. Additionally, the STEP study did not require functional decline for APS/BLIPS inclusion. We therefore hypothesised that STEP participants may present with lower symptom severity compared to PACE participants.

We compared baseline SANS and BPRS item scores between STEP and PACE participants (H_0_: STEP ≥ PACE vs. H_1_: STEP < PACE). Independent‐samples *t*‐tests indicated that STEP participants had significantly lower scores on avolition/apathy (*t* = −1.69, *p* = 0.046), attention (*t* = −2.98, *p* = 0.002), suspiciousness (*t* = −2.53, *p* = 0.006), hallucinations (*t* = −16.32, *p* < 0.001), and unusual thought content (*t* = −4.40, *p* < 0.001) compared to PACE participants. No significant group differences were observed for affective flattening, alogia, anhedonia/asociality, depression, suicidality, guilt, or conceptual disorganisation (all *p* > 0.05).

There was also a lower rate of transition to psychosis in the STEP study compared to previous PACE studies. The 12 months transition rate was about 34% in the PACE study versus 13.5% in STEP (McGorry et al. 2023; Yung et al. 2007). It could be that STEP participants were not as at high risk as the PACE participants, or participants in the STEP study may have been earlier in the course of illness than PACE participants and may not have developed inflammation yet. The removal of the requirement for a deterioration in functioning from the APS and BLIPS groups in the STEP study may also have resulted in the STEP cohort having lower levels of negative symptoms than the PACE cohort, as functioning is significantly associated with negative symptoms in the UHR group (Yung et al. 2019). This could have affected our ability to detect associations between inflammatory markers and negative symptoms, as these may be present only with higher levels of negative symptoms.

## 4 Discussion

In this study we assessed the association between inflammatory markers of A2M, IL‐6, CRP, sICAM‐1, sVCAM‐1 and suPAR and subtypes of negative symptoms. In general, we found no significant differences in inflammatory marker levels between negative symptom subgroups in this UHR sample. This is in contrast to previous systematic reviews and meta‐analyses in which positive associations have been found for pro‐inflammatory markers such as IL‐6 and CRP with negative symptoms in individuals with schizophrenia (Fernandez‐Sanchez et al. 2011; Goldsmith et al. 2019; Miller et al. 2011). After adjusting for multiple testing, higher sICAM‐1 levels were associated with lower odds of primary negative symptoms compared to no negative symptoms; however, this finding should be interpreted cautiously due to the small primary negative symptoms subgroup (*N* = 15) and was not observed consistently across other comparisons. Previous studies of sICAM‐1 in schizophrenia have generally shown that sICAM‐1 levels were increased in individuals with schizophrenia compared to controls (Meixensberger et al. 2021; Schwarz et al. 2000). A 2023 meta‐analysis found lower ICAM levels in first episode psychosis individuals and drug naïve patients with schizophrenia compared to controls (Li et al. 2023). There is also the suggestion that levels of sICAM‐1 increase after antipsychotic treatment (Schwarz et al. 2000), but this is also unclear (Sheikh et al. 2023).

Together with previous reports of lower sICAM‐1 levels in early psychosis and drug‐naïve patients, our findings are broadly consistent with some prior observations; however, given that this association was observed only in one comparison (primary negative vs. no negative) after adjustment for confounders and multiple testing, these findings should be interpreted with caution. Overall, existing evidence on sICAM‐1 in relation to negative symptoms remains inconsistent.

A combined total score of > 6 on the BPRS subscales of depression, suicidality and guilt (corresponding to greater than mild levels of symptoms) was one criterion for designating participants as having secondary rather than primary negative symptoms (the other criterion being greater than mild levels of positive symptoms). Given the association between depression and inflammatory markers (Gędek et al. 2025), we may have expected to find an association between secondary negative symptoms and such markers. However, no such association was found. We conducted a post hoc analysis to determine the level of depression, suicidality and guilt in the secondary negative symptom group (*n* = 54). The mean of these three subscales combined was 10.3 (SD 2.16). This corresponds to mild–moderate symptoms. This relatively low level of depressive symptoms may therefore explain the lack of association between secondary negative symptoms and inflammatory markers.

Age was associated with both primary negative symptoms and amotivation, with younger individuals showing higher odds of primary negative symptoms (compared to no negative symptoms) and amotivation. This finding is consistent with a previous study by Patel et al. which reported an association between younger age and negative symptoms (Patel et al. 2015). Smoking was associated with higher odds of secondary negative symptoms compared to no negative symptoms. This contrasts with some previous studies suggesting that smoking may be associated with lower negative symptoms in individuals with schizophrenia (Dalack et al. 1998). One possible explanation for these conflicting findings is that nicotine may temporarily improve mood and cognition (Benowitz 2009), which could be perceived as a reduction in negative symptoms. However, age and smoking were included in our models as confounders, and therefore this association should be interpreted cautiously and not as a primary finding of the study.

There are multiple possible explanations for the lack of association between negative symptoms and inflammatory markers in this study. First, the composition of the sample may play a role, as it may include individuals earlier in the course of illness or with a lower likelihood of progressing to psychosis compared to other UHR cohorts. Second, the assessment of negative symptoms presents challenges. Interrater reliability can be limited, as clinicians may differ in their interpretation of symptom severity, potentially leading to misclassification (Lindstrom et al. 1994). In addition, commonly used rating scales may not adequately distinguish between overlapping constructs such as depression and negative symptoms, contributing to diagnostic ambiguity (Kumari et al. 2017). These measurement limitations may have reduced our ability to detect associations with inflammatory markers. Third, only a small proportion of participants (19; 12.8%) met criteria for diminished expression. The limited size of this subgroup may have reduced statistical power, thereby limiting the ability to detect meaningful associations and warranting cautious interpretation of the findings.

Future research may benefit from incorporating complementary approaches to assessment, such as the Negative Symptoms Inventory Self‐Report (NSI‐SR), alongside clinician‐rated scales. Such measures may help better distinguish between primary and secondary negative symptoms and provide additional insight into patients' subjective experiences (Raugh et al. 2023).

Finally, it is also possible that there is no association between negative symptoms and inflammation in the UHR group, and that such a relationship may emerge only in later stages of schizophrenia, potentially reflecting processes related to disease progression (Williams et al. 2022).

## 5 Conclusion

In general, we found no association between inflammatory markers and subtypes of negative symptoms in this UHR sample. An inverse association between sICAM‐1 and primary negative symptoms was observed; however, this finding was limited to one comparison and should be interpreted with caution. Given that antipsychotic medications may alter immune function (Šafářová et al. 2025), future research in drug‐naïve early psychosis or UHR populations may provide clearer insight into the role of inflammatory biomarkers in psychosis. Longitudinal observational studies incorporating appropriate control groups are also needed to better understand the potential role of sICAM‐1 in psychosis.

## Funding

The authors have nothing to report.

## Data Availability

The data that support the findings of this study are available from the corresponding author upon reasonable request.
